# Effects of microbial fermentation on the anti-inflammatory activity of Chinese yam polysaccharides

**DOI:** 10.3389/fnut.2024.1509624

**Published:** 2025-01-06

**Authors:** Jinchu Yang, Yi Zheng, Yongfeng Yang, Zhenzhen Huang, Gangchun Sun, Renyong Zhao, Wen-Wen Zhou, Kit-Leong Cheong, Zichao Wang, Shouai Feng, Qiuling Wang, Meng Li

**Affiliations:** ^1^Technology Center, China Tobacco Henan Industrial Co., Ltd., Zhengzhou, China; ^2^School of Biological Engineering, Henan University of Technology, Zhengzhou, China; ^3^School of Chemistry and Chemical Engineering, Henan University of Technology, Zhengzhou, China; ^4^College of Food Science and Technology, Henan University of Technology, Zhengzhou, China; ^5^College of Biosystems Engineering and Food Science, Zhejiang University, Hangzhou, China; ^6^Guangdong Provincial Key Laboratory of Aquatic Product Processing and Safety, College of Food Science and Technology, Guangdong Ocean University, Zhanjiang, China; ^7^Technology Center, China Tobacco Guangxi Industrial Co. Ltd., Nanning, China; ^8^College of Tobacco Science and Engineering, Zhengzhou University of Light Industry, Zhengzhou, China

**Keywords:** Chinese yam, polysaccharide, *Lactobacillus plantarum* M616, fermentation, anti-inflammatory activity

## Abstract

In this study, Chinese yam polysaccharides (CYPs) were fermented using *Lactobacillus plantarum* M616, and changes in the chemical composition, structure, and anti-inflammatory activity of CYPs before and after fermentation were investigated. The carbohydrate content of *L. plantarum* M616-fermented CYP (CYP-LP) increased from 71.03% ± 2.75 to 76.28% ± 2.37%, whereas protein and polyphenol content were almost unaffected compared with those of the unfermented CYP (CYP-NF). The monosaccharide composition of CYP-NF included rhamnose, arabinose, galactose, glucose, and mannose in a molar ratio of 0.493:0.6695:0.9738:0.7655:12.4365. CYP-LP had the same monosaccharides as CYP-NF, but the molar ratio was 0.3237:0.3457:0.8278:2.5541:10.4995. Meanwhile, the molecular weight and polydispersity of CYP-LP, respectively, increased from 124.774 kDa and 6.58 (CYP-NF) to 376.628 kDa and 17.928, indicating a low homogeneity. *In vitro* antioxidant analysis showed that *L. plantarum* M616 fermentation had varying effects on CYP-LP against DPPH, ABTS, hydroxyl, and superoxide radicals. However, CYP-LP had superior anti-inflammatory activity to CYP-NF and is more effective in regulating superoxide dismutase, catalase, glutathione peroxidase, malondialdehyde, nitric oxide, tumor necrosis factor-*α*, interleukin-1β, and interleukin-6 release in lipopolysaccharide-induced RAW 264.7 macrophages. This study suggested that CYP-LP is a potential anti-inflammatory ingredient in drugs and functional food.

## 1 Introduction

Proper inflammation is beneficial and essential to the automatic defense of the body, but the persistence of inflammatory factors causes damage to body tissues and thus leads to the development of various diseases (1). Anti-inflammatory drugs are widely used to fight inflammation, but drug residues and bacterial resistance induced by the long-term use of anti-inflammatory drugs have seriously threatened human health (2). Apart from proper diet, personal and environmental hygiene, and proper exercise (3), consuming food containing anti-inflammatory compounds, especially dietary plant-derived anti-inflammatory substances, is essential. Li et al. (4) suggested that *Phaseolus lunatus* L. organic acids have anti-inflammatory activity and can be used in clinical efficacy studies. Sun et al. (5) demonstrated that *Nymphaea candida* polyphenols has excellent anti-inflammatory and cough-relieving properties. Zhao et al. (6) found that plant essential oils can be used to treat pain and inflammation, and Zhang et al. (7) found that alkaloids isolated from *Stemona tuberosa* Lour roots provide inflammatory protection to lipopolysaccharide (LPS)-damaged RAW 264.7 cells. Previously, we found that *Artemisia argyi* flavonoids have excellent antioxidant and anti-inflammatory activities (8, 9). Therefore, the exploitation and application of anti-inflammatory compounds from dietary plants have good prospects.

Polysaccharides are composed of more than 10 monosaccharides and have excellent physicochemical properties (10–12), and good anti-inflammatory activity (13, 14), especially polysaccharides from dietary plants. Yuan et al. (15) suggested that natural plant polysaccharides possess anti-inflammatory effects and can be used in drugs and functional food. Chen et al. (16) and Xie et al. (17) verified that *Astragalus membranaceus* and American ginseng polysaccharides can be used as anti-inflammatory ingredients. Huang et al. (18) suggested that pectic polysaccharides isolated from *Cucurbita moschata* Duch can reduce inflammatory responses and are potential functional food ingredients with anti-inflammatory properties. Thus, the application of dietary plant-based polysaccharides as anti-inflammatory ingredients is a good approach, especially polysaccharides extracted from medicinal and food-homologous plants.

Yam is the underground rhizome of *Dioscorea*, which is a medicinal and food-homologous plant (19). Yam polysaccharides have many bioactivities, including anti-inflammation activity. Lu et al. (20) found that yam polysaccharides alleviate DSS-induced ulcerative colitis in mice by inhibiting inflammation and modulating gut microbiota. Bai et al. (21) suggested that yam polysaccharides have intestinal anti-inflammatory activity. However, the low extractability and anti-inflammatory activity of yam polysaccharides limit their use as anti-inflammatory ingredients. Physical, chemical, and enzymatic methods might improve the extractability and bioactivity of yam polysaccharides (22–24), but simple, efficient, and green methods for enhancing these features simultaneously have not been discovered. Organic acids and enzymes produced by microorganisms not only can destroy plant cells and increase polysaccharide extractability but also modify polysaccharide structure (such as monosaccharide, molecular weight, functional group, glycosidic linkage, chemical bond, and spatial conformation) and bioactivity (25, 26).

In the present work, *Lactobacillus plantarum* M616 was used to ferment and modify Chinese yam polysaccharide (CYP), and the chemical composition and structural features of the CYPs before and after fermentation were analyzed. In addition, the antioxidant and anti-inflammatory activities of the CYPs were investigated. This work will provide a green and efficient method for improving the bioactivity of CYPs through microbial fermentation.

## 2 Materials and methods

### 2.1 Materials and microorganisms

Chinese yam (iron yam) was purchased from a local supermarket in Zhengzhou (Henan, China), which was provided by Wen County (Jiaozuo, Henan Province). *L. plantarum* M616 was provided by Dr. Yaoming Cui (Henan University of Technology, Zhengzhou, China). An MRS broth medium used for *L. plantarum* M616 activation and culture was purchased from Beijing Solarbio Science and Technology Co., Ltd. (Beijing, China). Ethanol, trichloromethane, 1-butanol, dialysis bag, and activated carbon used for the extraction and purification of yam polysaccharides were purchased from Beijing Solarbio Science and Technology Co., Ltd. (Beijing, China). Glucose, bovine serum albumin, gallic acid, potassium bromide, LPS, alicylic acid, ABTS, and other reagents used for detecting the chemical composition, structural features, and bioactivities of the yam polysaccharides were purchased from Beijing Solarbio Science &Technology Co., Ltd. (Beijing, China). Standard monosaccharides (fucose, rhamnose, arabinose, galactose, glucose, xylose, mannose, fructose, ribose, galacturonic acid, mannuronic acid, glucuronic acid, and mannuronic acid) used for detecting the monosaccharide of the yam polysaccharides were purchased from Sigma-Aldrich (Shanghai, China). The molecular weight and homogeneity of the yam polysaccharides were detected using SEC-MALLS-RI. Enzyme-linked immunosorbent assay (ELISA) kits used for determining superoxide dismutase (SOD), catalase (CAT), glutathione peroxidase (GSH-Px), malondialdehyde (MDA), nitric oxide (NO), tumor necrosis factor-*α* (TNF-α), interleukin-1β (IL-1β), and interleukin-6 (IL-6) factors were purchased from Beyotime Biotechnology (Shanghai, China).

### 2.2 Extraction of Chinese yam polysaccharides (CYPs)

Chinese yam was peeled, cut into small pieces, and crushed into paste with a grinder. One part of the pasted Chinese yam was placed in nine volumes (w/v) of deionized water, and the mixture was stirred magnetically at room temperature for 8 h. The resulting Chinese yam solution was collected and centrifuged at 8000 × *g* for 10 min for the removal of insoluble matter. Activated carbon was added to the supernatant with concentration of 1 g/100 mL, decolorized at 150 r/min overnight at room temperature, and centrifuged at 8000 × *g* for 10 min. The supernatant was concentrated to one-fifth at 60°C and 0.1 MPa and added to three volumes Sevag solution (trichloromethane: n-butyl alcohol = 3: 1). The mixture was shaken vigorously and then centrifuged at 10,000 × *g* for 10 min. Four volumes of alcohol were added to the supernatant and placed at 4°C overnight for the precipitation of the CYPs. Subsequently, the precipitated CYPs were re-dissolved in water and rotary evaporated at 60°C and 0.1 MPa. The CYP concentrate was de-salted through dialysis (molecular weight cut-off was 10.0 kDa) in deionized water for 48 h, and water was replaced every 4 h. Finally, a dialytic solution of unfermented CYP (CYP-NF) was collected and lyophilized.

Another other part of the pasted Chinese yam was added to nine volumes (w/v) of deionized water, peptone (1.0 g/L), yeast extract (1.0 g/L), MgSO_4_ (1.0 g/L), KH_2_PO_4_ (1.0 g/L), and K_2_HPO_4_ (1.0 g/L) and sterilized at 80°C for 60 min. Then, *L. plantarum* M616 grown to the logarithmic phase in the MRS broth medium was inoculated with 10% (v/v) volume and cultured at 35°C for 168 h. After fermentation, the fermentation broth was collected and filtered with eight layers of gauze for the removal of large sediments, and the filtrate was centrifuged at 10000 × *g* for 10 min. Then, extraction method for *L. plantarum* M616-fermented CYP was the same as that used for CYP-NF, and the extract was named CYP-LP.

### 2.3 Chemical composition analysis

Carbohydrate content in CYP-NF and CYP-LP was detected using the anthrone-sulfonic acid colorimetric method (27), polyphenol content was determined using the Folin–Ciocalteu method (28), and protein content was measured using the Coomassie Brilliant Blue method (29).

### 2.4 Monosaccharide and molecular weight detection

The monosaccharide composition, proportion, and molecular weight of CYP-NF and CYP-LP were detected by Shanghai Sanshu Biotechnology Co., Ltd. (Shanghai, China). The pretreatment and detection procedures of CYP-NF and CYP-LP were based on previously described methods (30).

### 2.5 Fourier transform infrared (FT-IR) spectroscopy analysis

Approximately 1 mg of freeze-dried CYP-NF or CYP-LP samples was mixed with 100 mg of potassium bromide, ground thoroughly, and pressed into tablets for detection using a FT-IR spectrometer (Nexus 470, Nicolet, USA). Each sample was detected three times and scanned through infrared spectroscopy from 500 cm^−1^ to 4,000 cm^−1^ with 1750 scanning points.

### 2.6 *In vitro* antioxidant activity of CYP-NF and CYP-LP

CYP-NF and CYP-LP were dissolved in deionized water to concentrations of 0.5, 1.0, 1.5, 2.0 and 2.5 mg/mL. Then, CYP-NF and CYP-LP solutions were filtrated through a 0.22 μm aqueous membrane. In vitro antioxidant activity against DPPH, ABTS, hydroxyl, and superoxide radicals were detected according to previously reported methods (30).

### 2.7 Toxicity analysis of CYP-NF and CYP-LP

The toxicity of CYP-NF and CYP-LP was detected using previously described methods (29). CYP-NF and CYP-LP were dissolved separately in Dulbecco’s modified eagle medium (DMEM) to concentrations of 5.0, 2.5, 1.25, 0.625, and 0.3125 mg/mL and filtrated through a 0.22 μm aqueous membrane. Their toxicity was evaluated with a CCK-8 kit on the basis of cell viability on RAW 264.7 macrophages.

### 2.8 Anti-inflammation analysis of CYP-NF and CYP-LP

Anti-inflammatory activity of CYP-NF and CYP-LP was detected according to previously reported methods (13). RWA 264.7 cells in the logarithmic growth phase were regulated to 2 × 10^5^ cell/mL with 0.25% (w/v) trypsin EDTA solution, and then 500 μL of RAW 264.7 cells were seeded into 12-well plates, incubated for 24 h, and treated with 1 μg/mL LPS for 24 h for the establishment of an inflammatory model. CYP-NF and CYP-LP were dissolved separately in DMEMs to concentrations of 5.0, 2.5, 1.25, 0.625 and 0.3125 mg/mL. CYP-NF or CYP-LP solution (100 μL) was added to each well and incubated for 24 h. The same amount of DMEM was used as the control. Then, the culture supernatant of the RAW 264.7 cells was collected for the detection of NO, TNF-*α*, IL-1β, and IL-6 levels according to the manufacturer’s protocols. The collected RAW 264.7 cells were cleaved with lyase and centrifuged. SOD, CAT, MDA, and GSH-Px activity were detected in the supernatant with ELISA kits.

### 2.9 Statistical analysis

All data were expressed as mean ± SD after three repeats, and the significance of the data was analyzed by one-way analysis of variance (ANOVA) performed with the methods reported previously (13).

## 3 Results and discussion

### 3.1 Chemical composition analysis

As shown in Table 1, the carbohydrate, protein, and polyphenol content in CYP-NF were 71.03% ± 2.75, 8.24% ± 0.19, and 0.26% ± 0.07%, respectively. After Chinese yam was fermented with *L. plantarum* M616 (CYP-LP), their content changed to 76.28% ± 2.37, 8.39% ± 0.26, and 0.25% ± 0.09%. Carbohydrate content increased, whereas protein and polyphenol content were basically unchanged. Similar results were obtained by previous studies, showing that microbial fermentation increases carbohydrate content in plant polysaccharides. Wang et al. (31) found that carbohydrate content in hot-water-extracted *Schisandra sphenanthera* fruit polysaccharide was 51.45% ± 1.78%, which increased to 63.22% ± 2.60% after *L. plantarum* CICC 23121 fermentation. Sakr (32) found that the carbohydrate content of fructan isolated from *Asparagus sprengeri* increased from 90.45% ± 0.28 to 94.11% ± 0.92% after *L. plantarum* DMS 20174 fermentation. However, some studied showed different results. Song et al. (33) found that *L. plantarum* CICC 24202 fermentation decreased carbohydrate content in Lanzhou lily polysaccharide from 93.56% ± 2.25 to 91.17% ± 1.93%. Shao et al. (34) and Tian et al. (35) found that carbohydrate content in Chinese yam and *Dendrobium officinale* was hardly affected by *Saccharomyces boulardii* and *Bacillus* sp. DU-106 fermentation. Carbohydrate content in plant polysaccharides might relate to fermentation strains and conditions, and high carbohydrate content might endow CYP-LP with enhanced bioactivity.

**Table 1:** Chemical composition and structural characteristics of Chinese yam polysaccharides before and after fermentation by *Lactobacillus plantarum* M616.

Chemical composition	CYP-NF	CYP-LP
Carbohydrate contents (%)	71.03 ± 2.75	76.28 ± 2.37
Protein content (%)	8.24 ± 0.19	8.39 ± 0.26
Polyphenol content (%)	0.26 ± 0.07	0.25 ± 0.09
**Monosaccharide composition (μg/mL)**		
Rhamnose	0.493	0.3237
Arabinose	0.6695	0.3457
Galactose	0.9738	0.8278
Glucose	0.7655	2.5541
Mannose	12.4365	10.4995
**Molecular weight (kDa)**		
Weight-average molecular weight (M_w_)	124.774	376.628
Number-average molecular weight (M_n_)	18.963	21.008
Polydispersity (M_w_ / M_n_)	6.58	17.928

### 3.2 Monosaccharide analysis

Monosaccharides may affect bioactivity and functions of polysaccharides by influencing their electrification and functional groups (36). Table 1 shows that CYP-NF composed rhamnose, arabinose, galactose, glucose, and mannose in a molar ratio of 0.493:0.6695:0.9738:0.7655:12.4365. CYP-LP contained the same monosaccharides in a molar ratio of 0.3237:0.3457:0.8278:2.5541:10.4995 after *L. plantarum* M616 fermentation. Additionally, Guo et al. (37) and Li et al. (38) found that yam polysaccharides contain uronic acid. However, it was not detected in CYP-NF and CYP-LP. Huang et al. (39) and Wan et al. (40) found *Lactobacillus fermentum* fermentation only affected the molar ratio of polysaccharides isolated from longan pulp and carrot pulp, but their monosaccharide types were not influenced. Yang et al. (41) found that *Lactobacillus casei* fermentation did not affect the monosaccharide composition of *Polygonatum kingianum* polysaccharides. These results were similar to those in the present work. However, Gao et al. (42) and Song et al. (33) found that *L. plantarum* fermentation decreased the monosaccharide types of *Momordica charantia* L. and Lanzhou lily polysaccharides. Meanwhile, Huang et al. (43) suggested that monosaccharide type change in the longan pulp polysaccharides is related to *L. plantarum* fermentation time.

### 3.3 Molecular weight

Molecular weight may affect the bioactivity of polysaccharides by influencing their morphology, size, spatial configuration, absorption, and utilization rates (44, 45). As shown in Table 1, the weight-averaged and number-averaged molecular weight of CYP-NF were 124.774 and 18.963 kDa, respectively, and its polydispersity was 6.58. After fermentation with *L. plantarum* M616, the weight-averaged molecular weight, number-averaged molecular weight, and polydispersity of CYP-LP increased to 376.628 kDa, 21.008 kDa, and 17.928, respectively. These results indicated that *L. plantarum* M616 fermentation increased molecular weight and reduced homogeneity of CYP-LP. On the one hand, enzymes and organic acids secreted by *L. plantarum* M616 hydrolyzed Chinese yam polysaccharides with small molecular weight into oligosaccharides or monosaccharides, thus increasing CYP-LP molecular weight (46). On the other hand, *L. plantarum* M616 fermentation increased the number of hydroxyl groups in Chinese yam polysaccharides, then stretching vibration of hydroxyl groups caused aggregation of polysaccharide molecules and the increased CYP-LP molecular weight (47). Last but not least, the polysaccharide synthase secreted by *L. plantarum* M616 polymerized small molecular weight polysaccharides into large molecular weight ones via enzymatic polymerization, thus increasing the molecular weight of CYP-LP (48, 49). Tian et al. (35) found that the molecular weight of *Dendrobium officinale* polysaccharide increased from 4.92 × 10^5^ Da to 5.21 × 10^5^ Da after *Bacillus* sp. DU-106 fermentation. Liang et al. (46) found that the molecular weight of *Lentinus edodes* polysaccharide increased from 1.16 × 10^4^ Da to 1.87 × 10^4^ Da after *Lactobacillus fermentum* 21,828 fermentation. In general, enzymes and organic acids secreted by microorganisms might reduce the molecular weight of polysaccharides by breaking glycosidic linkages (25). Yang et al. (41) found that *Lactobacillus casei* fermentation decreased the molecular weight of *Polygonatum kingianum* polysaccharides from 50–650 kDa to 2–100 kDa. Sakr (32) found that *L. plantarum* DMS 20174 fermentation reduced the molecular weight of *Asparagus sprengeri* fructan from 1770 Da to 1,229 Da. Meanwhile, molecular weight is affected by fermentation strains and conditions. Yang et al. (50) suggested that the molecular weight of *Sargassum fusiforme* polysaccharides was almost unaffected by *Lactobacillus* fermentation. He et al. (51) and Huang et al. (43) verified that the molecular weight of litchi pulp and longan pulp polysaccharides decreased and then increased with the extension of *Lactobacillus* fermentation time. Effect of microbial fermentation on the molecular weight of polysaccharides might be one of the focuses of future research.

### 3.4 Fourier transform infrared (FT-IR)

**Figure 1:**
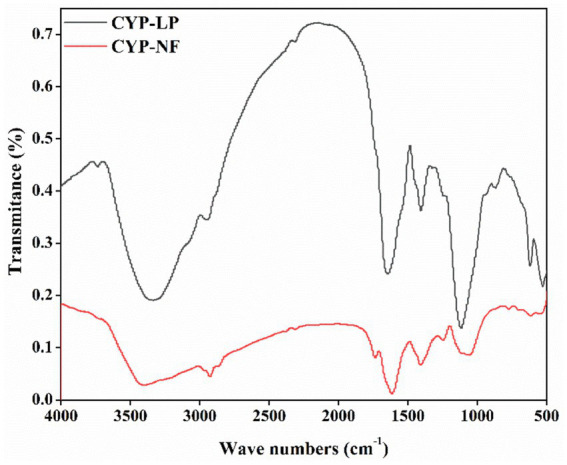
FT-IR spectra of Chinese yam polysaccharides before and after fermentation by *Lactobacillus plantarum* M616, and the mean spectral is generated from n = 3.

The types and amounts of functional groups affect the bioactivity of polysaccharides (25, 52), and the structural features of yam polysaccharides influenced by *L. plantarum* M616 fermentation were analyzed by FT-IR. As shown in Figure 1, peaks between 3,400 and 3,200 cm^−1^ might relate to the intermolecular H-bridge of OH groups and OH stretching, and peaks between 3,000 and 2,900 cm^−1^ might relate to CH_2_ antisymmetric stretching. They were the characteristic absorption peaks of polysaccharides (30). Peaks between 1800 and 1700 cm^−1^ might relate to COOH groups or C=O stretching from acetyl, and peaks between 1,600 and 1,400 cm^−1^ might relate to CH_2_ symmetric ring stretching or to the vibration of CH_2_ scissors in CYP-NF and CYP-LP (28). Peaks between 1,400 and 1,100 cm^−1^ might relate to OH in-plane deformation, C-O-C antisymmetric stretching and C-O stretching (13), and peaks between 900 and 500 cm^−1^ might relate to C-anomeric group stretching and pyran ring stretching (27). Figure 1 also shows that CYP-NF and CYP-LP had similar FT-IR spectra but different peak height, indicating that they had the same types of functional groups and *L. plantarum* M616 fermentation did not influence the functional groups and main structure of the yam polysaccharides but affected the amounts of the functional groups.

### 3.5 *In vitro* antioxidant activity

**Figure 2:**
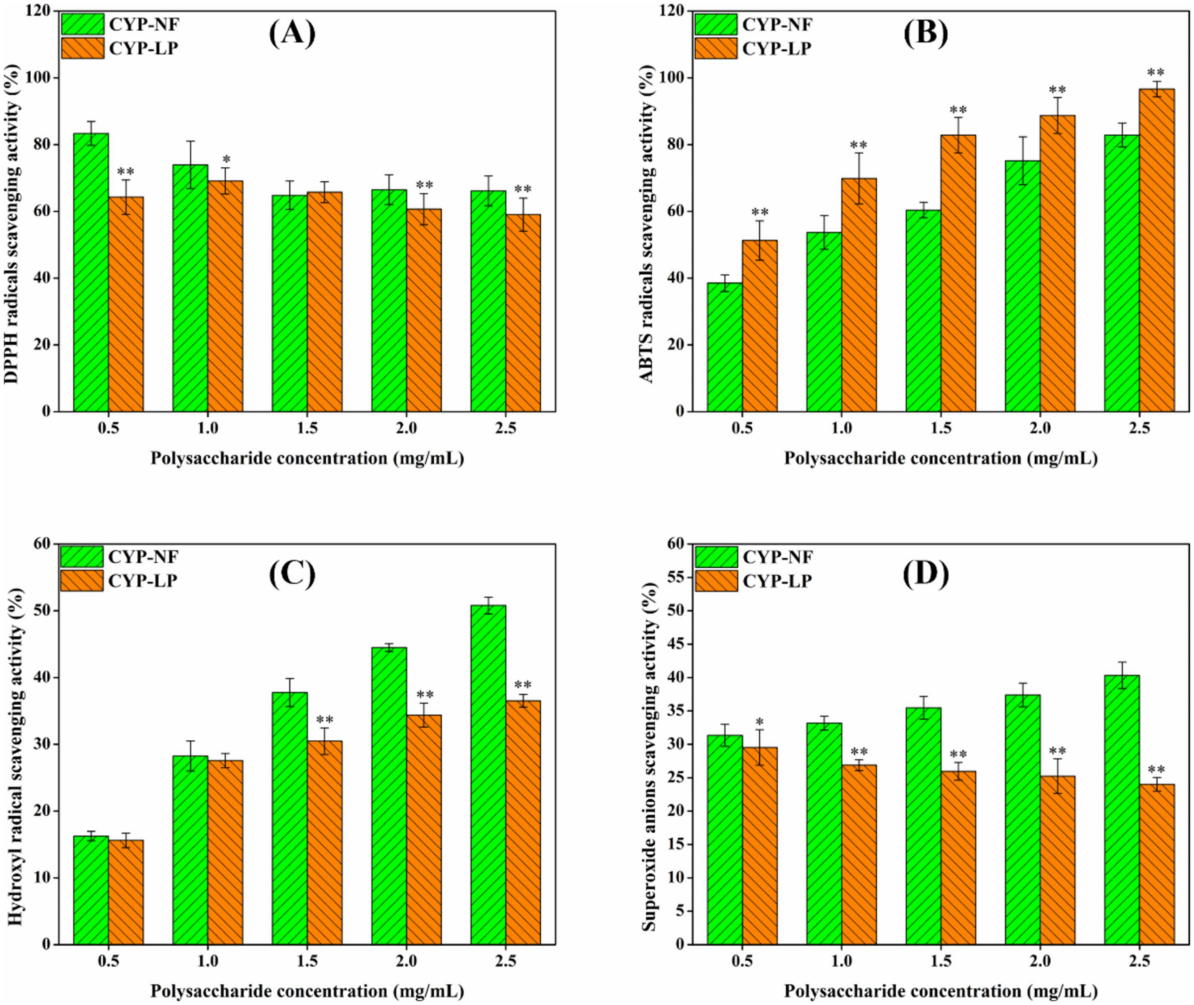
*In vitro* antioxidant activity of CYP-NF and CYP-LP against DPPH **(A)**, ABTS **(B)**, hydroxyl **(C)** and superoxide **(D)** radicals. ^*^ *p* < 0.05, ^**^ *p* < 0.01 as compared to CYP-NF.

Excessive oxygen free radicals can attack and damage biomacromolecules in the body, thus inducing inflammation and various diseases (53). As shown in Figure 2A, the scavenging effect of CYP-NF against DPPH radicals decreased slightly from 83.32 to 66.11% as concentration increased from 0.5 mg/mL to 2.5 mg/mL. However, the scavenging effect of CYP-LP against DPPH radicals was basically unchanged. Figures 2B,C show that the scavenging effects of CYP-NF and CYP-LP against ABTS and hydroxyl radicals increased as concentrations increased from 0.5 mg/mL to 2.5 mg/mL. CYP-LP showed higher activity against ABTS radicals but lower activity against hydroxyl radicals than CYP-NF. Figure 2D shows that the scavenging effect of CYP-NF against superoxide radicals slightly increased with concentration, and CYP-LP showed an opposite trend. In general, microbial fermentation might increase the extraction rate and carbohydrate content, and modify polysaccharide structure (such as reducing molecular weight and changing monosaccharide composition, and so on), thus improving the antioxidant activity of polysaccharides (25, 26). Yu et al. (54) found that the scavenging effects of jackfruit polysaccharide against DPPH and ABTS radicals were enhanced after fermentation by *L. plantarum* FM 17. Yang et al. (41) found that the DPPH radical-scavenging activity and total reducing power capacity of *Polygonatum kingianum* polysaccharide improved after *Lactobacillus casei* fermentation. However, in some cases, changes in chemical composition and structural features (including the decrease of bioactive functional groups and spatial structures, and so on) after microbial fermentation may reduce the antioxidant activity of polysaccharides (25, 26). Wang et al. (55) verified the scavenging effects of Lvjian okra polysaccharide against DPPH, ABTS and hydroxyl radicals were reduced after *L. plantarum* fermentation. Song et al. (33) found that the antioxidant activity of Lanzhou lily polysaccharide against hydroxyl radicals was reduced by *L. plantarum* fermentation. Therefore, the effect of *L. plantarum* M616 fermentation on the structure and antioxidant activity of yam polysaccharides will be further analyzed in our future studies.

### 3.6 Toxicity analysis

**Figure 3:**
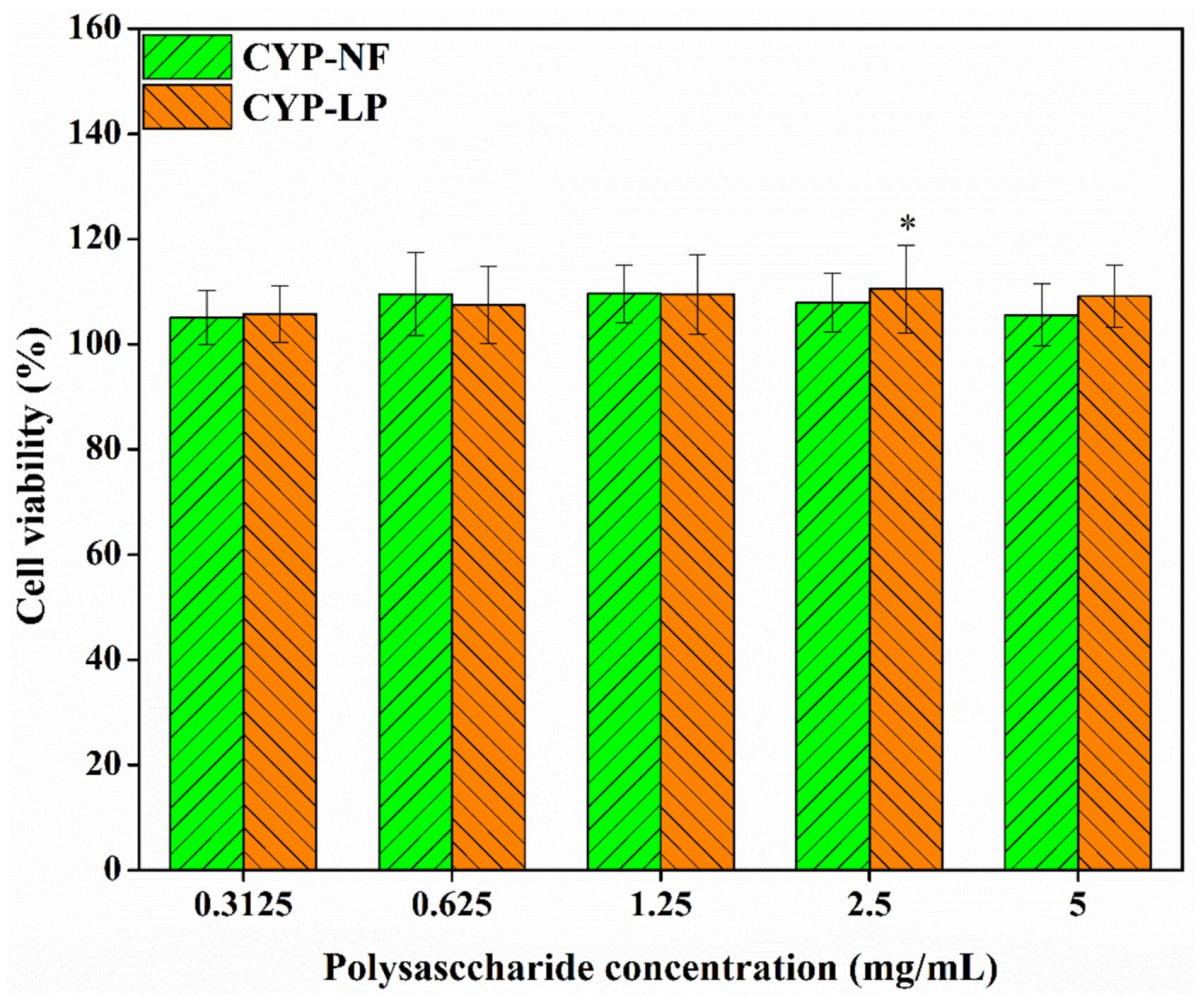
Toxicity analysis of CYP-NF and CYP-LP. ^*^ *p* < 0.05, ^**^ *p* < 0.01 as compared to 100% cell activity.

Chinese yam is a medicinal and food-homologous plant (19, 56), and yam polysaccharides have good biosafety. As shown in Figure 3, cell viability was maintained at 100–105% after the addition of different concentrations of CYP-NF and CYP-LP, suggesting their biosafety. As biomacromolecules, polysaccharides have good biosafety. Shao et al. (57) found that yam peel polysaccharide exerts an effect that promote the proliferation of RAW 264.7 cells. Li et al. (58) found that *Dioscotea opposite* polysaccharides have no toxicity on RAW 264.7 macrophages. Meanwhile, the safety of plant polysaccharides modified by microbial fermentation has been verified. Wang et al. (55) demonstrated that *L. plantarum*-fermented Lvjian okra polysaccharides have no toxic effects on RAW 264.7 macrophages. Tian et al. (35) indicated that *Bacillus* sp. DU-106-fermented *Dendrobium officinale* polysaccharides promote RAW264.7 cell proliferation without exerting cytotoxic effects.

### 3.7 Anti-inflammatory activity

**Figure 4:**
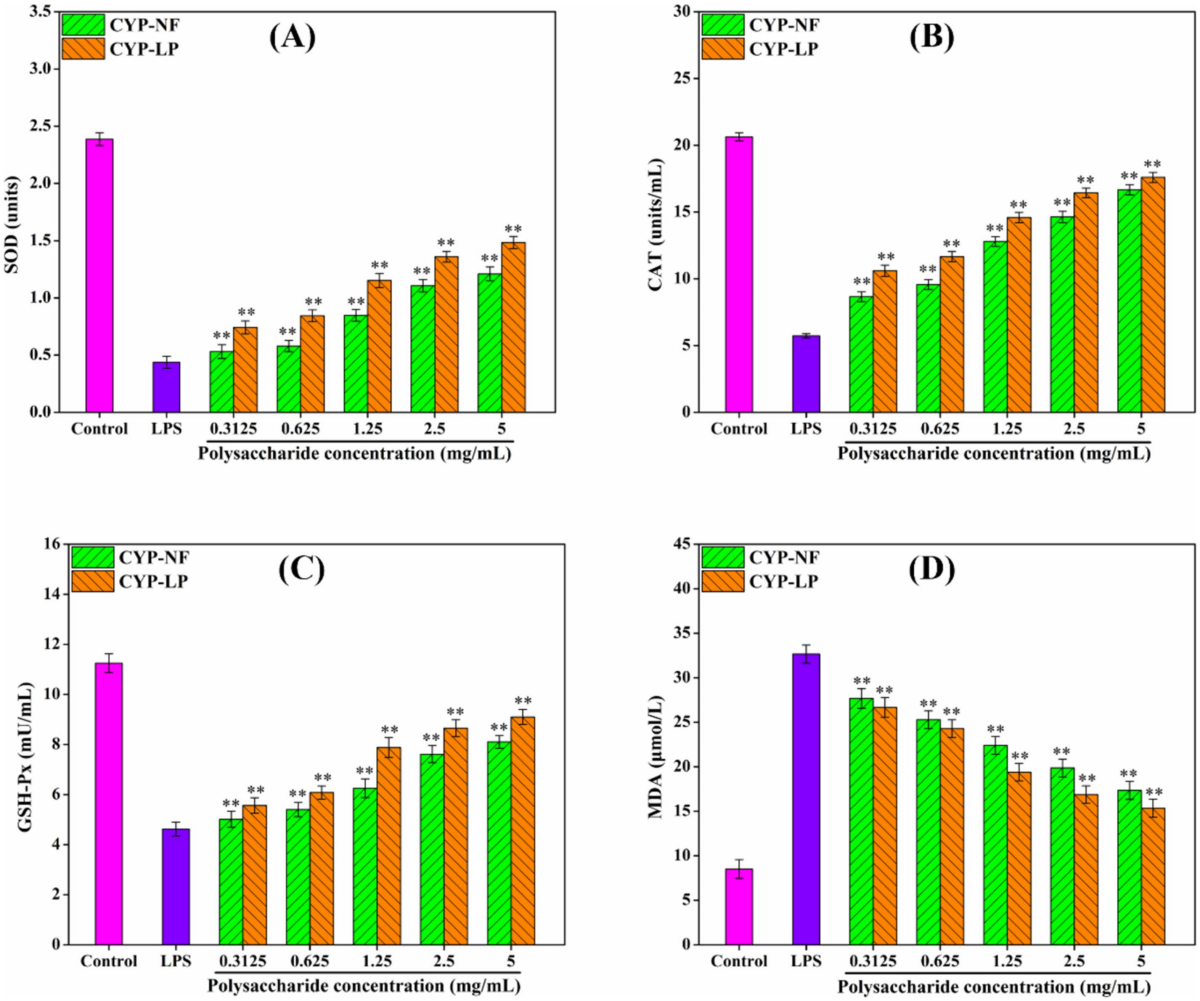
Effect of CYP-NF and CYP-LP on SOD **(A)**, CAT **(B)**, GSH-Px **(C)** and MDA **(D)** activities in LPS-induced RAW 264.7 macrophages. ^*^ *p* < 0.05, ^**^ *p* < 0.01 as compared to control group.

**Figure 5:**
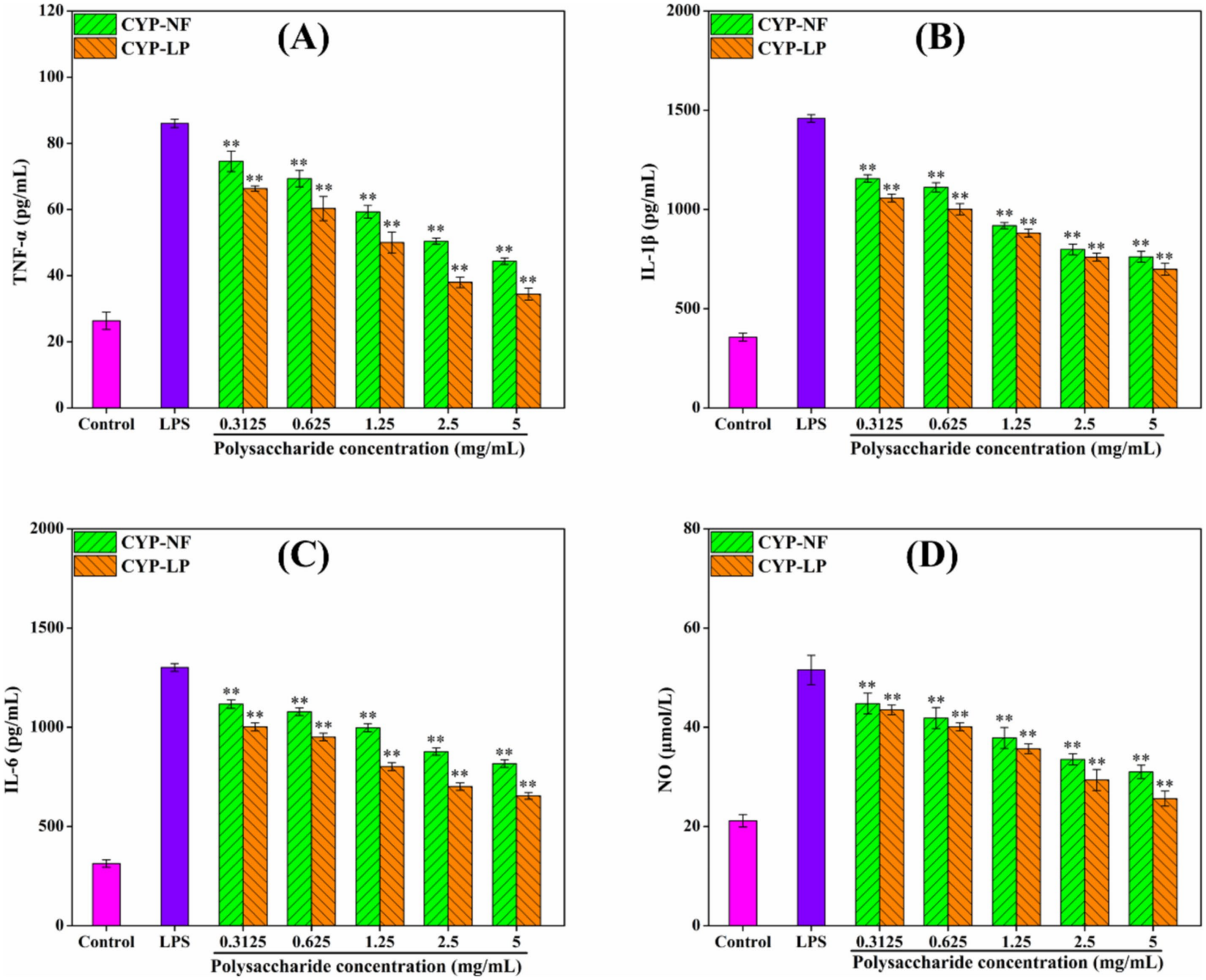
Effect of CYP-NF and CYP-LP on TNF-*α* **(A)**, IL-1β **(B)**, IL-6 **(C)** and NO **(D)** activities in LPS-induced RAW 264.7 macrophages. ^*^ *p* < 0.05, ^**^ *p* < 0.01 as compared to control group.

The persistence of inflammatory factors will damage body tissues, thus leading to development of various diseases (1, 59). Figure 4 shows that CYP-NF and CYP-LP increased SOD, CAT, and GSH-Px levels in LPS-induced RAW 264.7 macrophages and reduced MDA formation. CYP-LP had a higher effect than CYP-NF. Meanwhile, Figure 5 shows similar trend. CYP-NF and CYP-LP reduced the levels of TNF-*α*, IL-1β, IL-6, and NO in LPS-induced RAW 264.7 macrophages, and CYP-LP had more enhancing effects than CYP-NF. Although *L. plantarum* M616 fermentation had a different effect on the *in vitro* antioxidant activity of CYP-LP, the lower homogeneity of CYP-LP indicated the presence of fractions with different molecular weight, which might have enhanced anti-inflammatory activity (21). Meanwhile, change in monosaccharide composition (such as galactose) may afford CYP-LP enhanced anti-inflammatory activity (36). Improvement in the anti-inflammatory activities of plant polysaccharides through microbial fermentation has been verified. Tang et al. (60) suggested that *Limosilactobacillus reuteri* CCFM8631 fermentation enhances *Dendrobium officinale* polysaccharides to reduce NO and IL-6 secretion. Zhang et al. (61) found that *L. plantarum* NCU116 fermentation improved *Asparagus officinalis* polysaccharide to inhibit TNF-*α* and IL-1β expression, and reinforced antioxidant systems (T-AOC, SOD, CAT, and MDA) in mice with liver injuries. Additionally, Li et al. (62) demonstrated that *Lactobacillus* fermentation enhanced the alleviating effect of *Nostoc commune* Vauch. polysaccharides in cadmium-injured mice by increasing the activity of antioxidant enzymes (SOD, GSH, and GSH-Px) and inhibiting cytokines levels (IL-6, IL-1β, TNF-α, and IL-18). However, Chen et al. (63) reported that yeast fermentation had little effect on the anti-inflammatory activity of *Dendrobium officinal* polysaccharides. Meanwhile, microbial fermentation might decrease the anti-inflammatory activity of polysaccharides. Wang et al. (55) found that *L. plantarum* P158 fermentation decreased the immunomodulatory ability of Lvjian okra polysaccharide to stimulated the secretion of NO and IL-6.

## 4 Conclusion

Carbohydrate content increased from 71.03% ± 2.75% (CYP-NF) to 76.28% ± 2.37% (CYP-LP) after *L. plantarum* M616 fermentation. Meanwhile, CYP-LP had higher molecular weight and changed molar ratio compared to CYP-NF. However, *L. plantarum* M616 fermentation endowed CYP-LP with different antioxidant activities *in vitro*, and CYP-LP showed better anti-inflammatory activity than CYP-NF. Overall, the present study not only offers a good reference for the green and efficient modification of plant polysaccharides through microbial fermentation but also offers an excellent strategy for producing plant-based functional beverages. Unfortunately, the effects of *L. plantarum* M616 fermentation on the physicochemical properties (including viscosity, water holding capacity, suspension and thickening abilities) of CYP-LP were not analyzed. These effects will be the focus of future research.

## Data Availability

The original contributions presented in the study are included in the article/supplementary material, further inquiries can be directed to the corresponding authors.
